# Investigation of the synthesis, gelation potential, and drug-loading capacities of two novel amides

**DOI:** 10.3389/fchem.2024.1369542

**Published:** 2024-05-10

**Authors:** Deniz Bariş Cebe, Elif Kötekoğlu

**Affiliations:** Department of Chemistry, Faculty of Arts and Science, Batman University, Batman, Türkiye

**Keywords:** biocompatible materials, low-molecular weight organogel, L-isoleucine, organogelators, tetraamide compounds

## Abstract

This study consists of four steps. In the first, two different biocompatible organogelators were synthesized, starting with the *L-*isoleucine amino acid to obtain amide compounds. In the second step, the gelation potential of synthesized organogelators with fatty acid esters and organic solvents was investigated. These esters were chosen as gelation liquids due to their biocompatibility and also their penetration-enhancing properties when the drug is administered via the skin. After the minimum gel concentrations (MGCs) of the organogelators were determined, the melting point of gel *T*_g_ was found, and then, Δ*H*_g_ gelation enthalpy values were found by means of the Van’t Hoff equation. In addition to the gelation abilities and capacities of the organogelators being thus synthesized, their thermal stabilities were also determined. In the third stage of the study, the network which occurred during the formation of the gels was screened by an SEM device, and their characterizations were determined. In the study's fourth stage, the gels were loaded with ibuprofen and naproxen—known for their non-steroidal anti-inflammatory and analgesic effects—and their drug-loading capacities were thus determined.

## 1 Introduction

Gels are structures with an immobilized outer solvent and semi-solid formulation which are present within a network as nonpolar organogels or polar hydrogels (Vintiloiu and Leroux, 2008). The main forces in the combinations of gelators (self-assembly) are inter-cellular, non-covalent transaction types such as hydrogen-bound, π–π aggregation, Van der Waals interactions, coordination attractions, and load transfer interactions*.* These non-covalent interactions form super-molecular structures with nano-cavities. Gels are then formed in these cavities due to the solvent trapped in them (Suzuki et al., 2008). Low-molecular weight gelators (LMWGs) have become a focus of interest, owing to their potential applications as new soft materials and their unique properties (Terech and Weiss, 1997; Terech et al., 2002; Estroff and Hamilton, 2004; Sangeetha and Maitra, 2005). Gels have wide usage in daily life from food to medicine and biomaterials and to cosmetics and other technologies (Abdallah and Weiss, 2000a). Gelation requires gelling liquid and a small quantity of solid matrixes. Gels, which are smart materials, are quite interesting since they are sensitive to a wide variety of chemical and physical triggers (Fairman and Akerfeldt, 2005).

The biodegradability, biocompatibility, and moderate physical and chemical properties of LMWGs show that they have a significant place in biomedical applications and medication (Zhang et al., 2003; Zhou et al., 2005). An important challenge is the frequent use of organic compounds, nanomaterials, and secondary metabolites found in plant and fungal extracts to develop and design new drugs and gelators with a highly selective effect against viral, bacterial, and venereal diseases (Gunashova, 2022; Nasibova, 2022; Pashazade, 2022; Binate and Ganbarov, 2023). In providing an environment for improving the stability of capsuled drug molecules, LMWGs contribute to the prevention of enzymatic degradation during medication. The self-formation of gels during the production of medication-loaded material is another advantage of their use (Branco et al., 2009). Many instances of LMWGs being administered as a novel pharmaceutical medium have been documented recently (Kobayashi et al., 2002; Heeres et al., 2003). Nonetheless, the application of LMWGs has been planned for gelling organic solvents, and examining the relationship between the structure of gelators and their gelling abilities has been envisaged (Abdallah and Weiss, 2000b; Esch and Feringa, 2000; Gronwald and Shinkai, 2001).

Gels are classified according to the bonds that keep the molecules within the gelator network. Although molecules in chemical gels are kept together with the help of covalent bonds, the physical gels cohere with the help of weaker physical attraction forces such as van der Waals interactions and hydrogen bonds. Lower hydration is present in the medicinal organogels that are transferred between chains after being solved in polymer. Cross-linking leads to the increased hydrophobicity of gels, yet a decrease in medication diffusion speeds it up. When used in concentrations smaller than 15%, gelators could be exposed to physical and chemical interactions such that they can intertwine as fiber structures which cause the formation of a three-dimensional network structure which prevents the flow of outer non-polar phase. Some thermo-sensitive molecule-forming gels in the body’s temperature environment can be given as parenteral in a convenient liquid. Organogels, with the property of thermo-reversibility, attract significant attention because of their potential use in controllable drug delivery systems. Not only the sensitivity to heat but also the sensitivity to humidity of organogels has been investigated in developing these systems. Low-molecular weight organogelators are mostly composed of tiny molecules, as opposed to hydrogels, which are often polymeric gelators. Various organogel-based formulations have been designed as medication for different deliveries of bio-active agents (Vintiloiu and Leroux, 2008). One of the difficulties encountered in developing low-molecular weight gelators is stabilizing the formed gel—that is, preventing the semi-stabilized gel from becoming crystalized. Due to the high cost and lack of large-scale production of lecithin and other natural-origin raw materials, synthetic amino acid-based organogelators have replaced these natural raw materials (Gupta et al., 2011; Tarun et al., 2011). In this study, two new low-molecular weight tetraamide compounds were synthesized, beginning with L-isoleucine, and their gelation potential was investigated in order to characterize the gels that were generated. Ibuprofen and naproxen were added to the gels to allow their loading capabilities to be ascertained.

## 2 Materials and methods

### 2.1 Apparatus for measurements

Infrared spectra were recorded on a MATTSON Model 1000 Spectrophotometer. ^1^H NMR (400 MHz) and ^13^C NMR (100 MHz) spectra were recorded on a Bruker AV-400 High-Performance Digital FT- NMR Spectrometer. Elemental analyses C, H, and N were performed using Thermo Scientific Flash 2000 model apparatus. The SEM observations were conducted using a FEI Quanta 250 FEG field emission scanning microscope.

### 2.2 Synthesis

**FIGURE 1:**
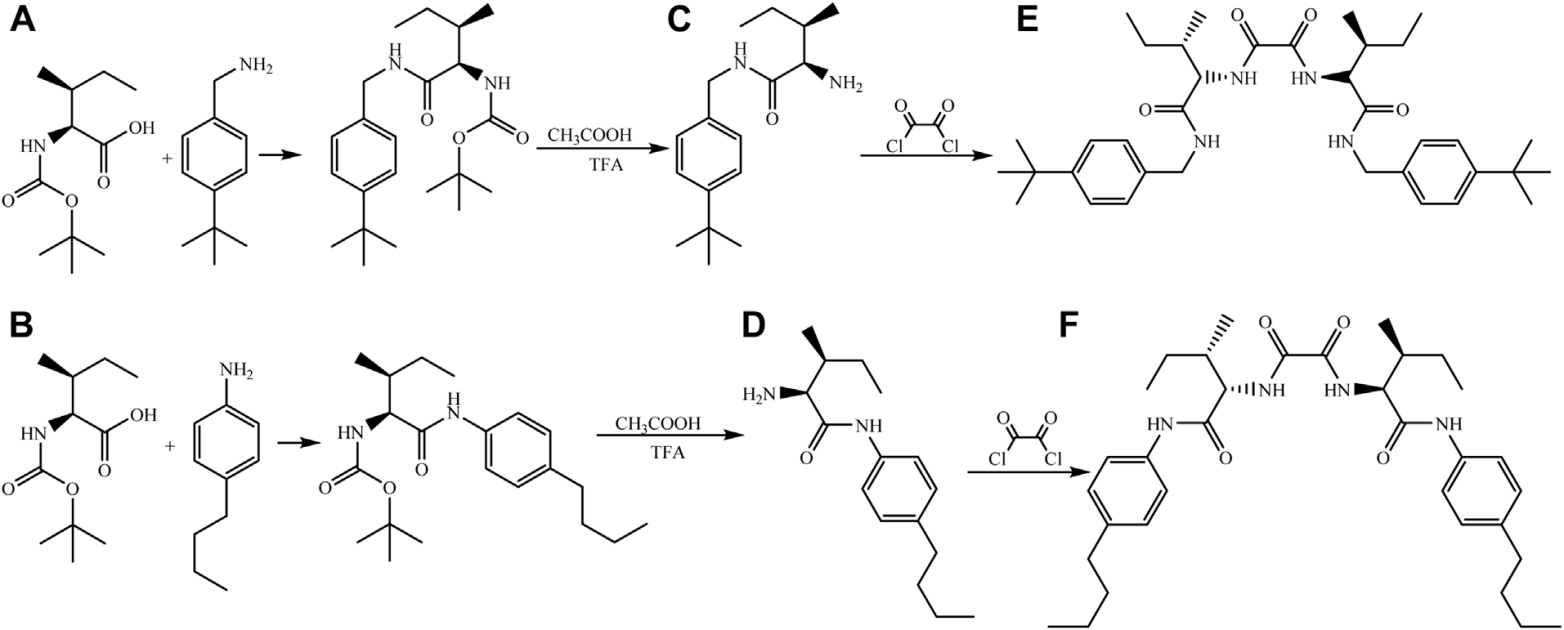
Synthesis of amide compounds (organogelators). Synthesis of organogelator E starting from organic compound A. Synthesis of organogelator F starting from organic compound B.

In the first stage of this study, two new *C*_2_-symmetric chiral tetraamide compounds were synthesized, starting with *L*-isoleucine. N-Boc amine compounds (A, B) were synthesized by the reaction of N-boc-*L*-isoleucine with corresponding amines. Deprotection of the boc group gave compounds C and D, which bear the free amine group (Sunkur et al., 2021). Then, the reaction of C and D with oxalyl chloride at room temperature produced two new *C*_2_-symmetric chiral tetraamide compounds (E, F) by quantitative yields (Figure 1).

In the second stage of the study, the gelation properties of these organogelators were investigated. Fatty acids (isopropyl and ethyl esters of palmitic, lauric, and myristic acid) and widely used solvents (liquid paraffin, anisole, xylene, toluene, diethyl glycol, n-dodecane, and chloroform)—the biocompatible liquids utilized in the drug and cosmetics industries—were used as gelation solvents. The gelation potential of organogelators with these solvents was examined (determination of minimum gelation concentration, measurement of *T*_g_, and determination of gel–sol transition enthalpy (∆*H*_g_).

#### 2.2.1 Synthesis of organogelator 1 (N^1^,N^2^-bis(2S,3S)-1-((4-(tert-butyl)benzyl)amino)-3-methyl-1-oxopentan-2-yl)oxalamide)

**FIGURE 2:**
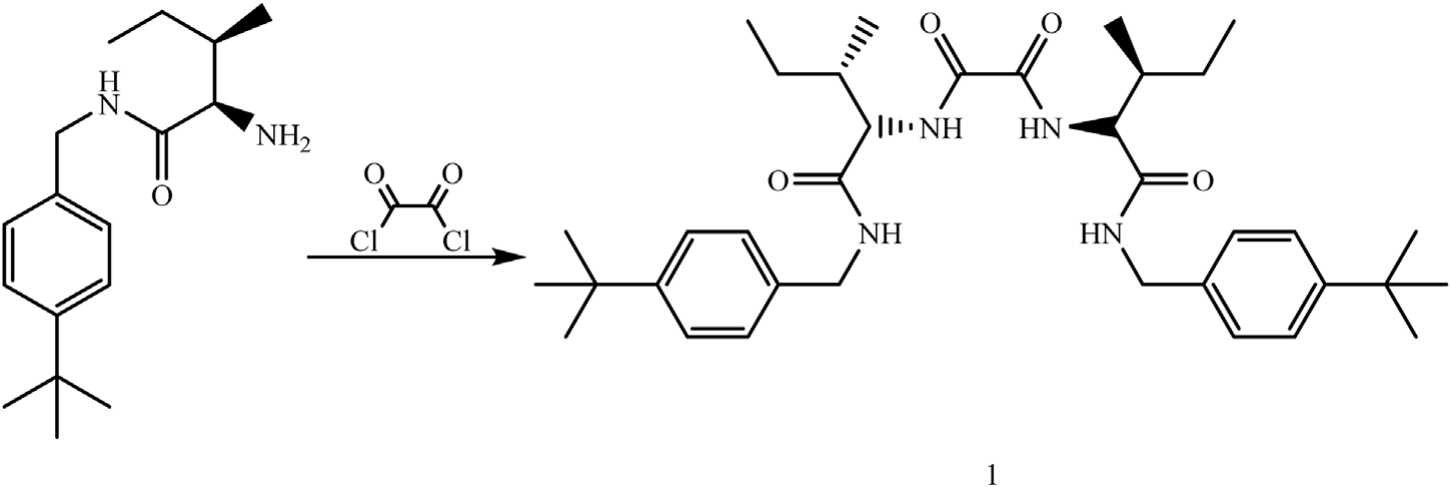
Synthesis of organogelator 1.

A solution of oxalyl chloride (0.211 g, 1.66 mmol) was added to a solution of (2*R*,3*R*)-2-amino-*N*-(4-(*tert*-butyl)benzyl)-3-methylpentanamide (0.92 g 3.33 mmol) in dry THF (20 mL) dropwise at 0 °C under the Ar atmosphere. The mixture was stirred at room temperature for 1 h. After the reaction was stopped, the mixture was extracted with 1 N HCl (100 mL × 2), 10% NaHCO_3_ (100 mL × 2), and distilled water (100 mL × 2), respectively. The organic phase was dried on MgSO_4_, filtered, and THF was evaporated to obtain white solids as a pure product (1.0 g, 100%) (Figure 2). Mp: 215°C (decomp.). ^1^H NMR (DMSOd_6_): δ = 1.20 (m, 30H, CH_3_), 3.04 (m, 8H, CH_2_), 4.23 (m, 4H, CH), 7.15–7.34 (m, 8H, ArH), 8.31 (d, 2H, NH), and 8.66 (t, 2H, NH). ^13^C NMR (CDCl_3,_ 100 MHz) δ (pmm): 8.9, 11.3, 24.9, 31.6, 34.6, 58.1, 125.5, 127.6, 149.8, 159.5, and 170.3. FTIR (cm^-1^): 3,285–3,279, 3,080, 2,963, 1,652, 1,544, 1,477, 1,397, 1,172, and 1,036. Elemental analysis calculated (%) for C_36_H_54_N_4_O_4_ was C: 71.25, H: 8.97, and N: 9.23. The value found was C: 71.32, H: 8.89, and N: 9.20.

#### 2.2.2 Synthesis of organogelator 2 (N^1^,N^2^-bis(2S,3S)-1-((4-butylphenyl)amino)-3-methyl-1-oxopentan-2-yl)oxalamide)

**FIGURE 3:**
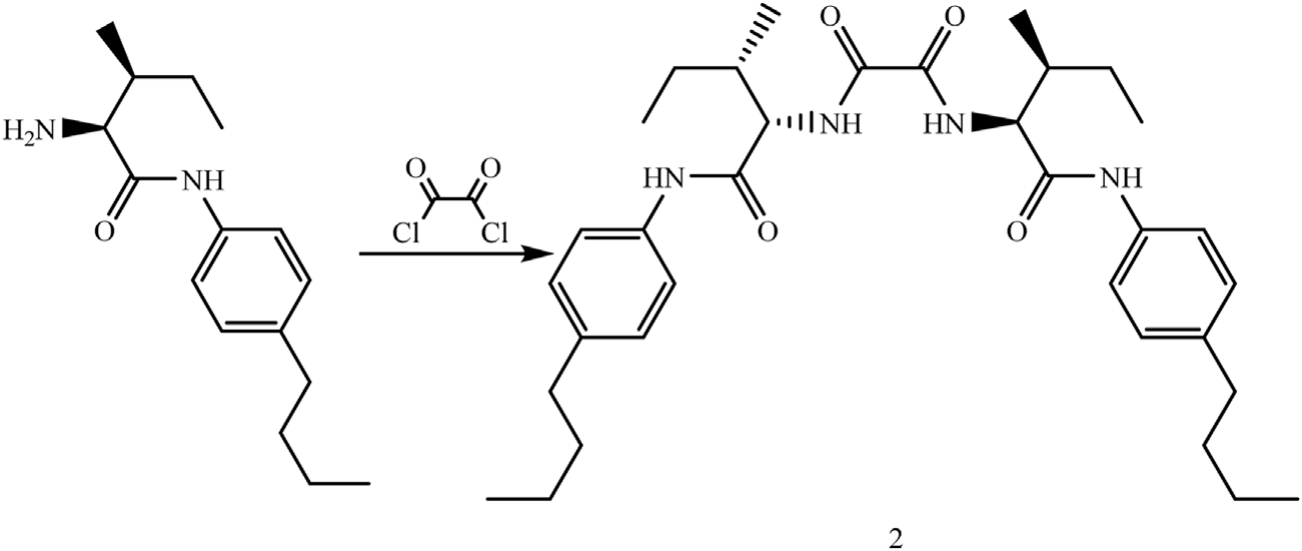
Synthesis of organogelator 2.

A solution of oxalyl chloride (0.174 g, 1.37 mmol) was added to a solution of (2*S*,3*S*)-2-amino-*N*-(4-butylphenyl)-3-methylpentanamide (0.72 g, 2.75 mmol) in dry THF (20 mL) dropwise at 0 °C under Ar atmosphere. The reaction was stirred at room temperature for 1 h. After the reaction was stopped, the mixture was extracted with 1 N HCl (100 mL × 2), 10% NaHCO_3_ (100 mL × 2), and distilled water (100 mL × 2). The organic phase was dried on MgSO_4_, filtered, and THF was evaporated under reduced pressure with a rotary evaporator to obtain white solids as a pure product (0.79 g, 100%) (Figure 3). Mp: 198°C (decomp.). ^1^H NMR (DMSOd_6_): δ = 0.89–1.21 (m, 18H, CH_3_), 1.50–1.70 (m, 12H, CH_2_), 2.50 (s, 6H, CH_2_ and CH), 3.36 (m, 2H, CH), 7.10–7.56 (m, 8H, ArH), and 8.44 (d, 4H, NH). ^13^C NMR (CDCl_3,_ 100 MHz) δ (pmm): 11.6, 14.5, 15.6, 22.1, 33.6, 34.7, 37.2, 65.4, 120.0, 128.9, 136.5, 138.2, 159.7, 167.1, and 169.2. IR (cm-1): 3,264 (-C-H aromatic), 3,078(-C-H aromatic) 2,979–2,939 (-C-H aliphatic group), 1,654(-C=H aromatic), 1,525 (-C=N aromatic), 1,477 (N-H bound to amine), and 1,172–1,036 (C-O- R-OR). Elemental analysis calculated (%) for C_34_H_50_N_4_O_4_ was C: 70.56, H: 8.71, and N: 9.68. The value found was C: 70.61, H: 8.81, and N: 9.64.

### 2.3 Determination of minimum gel concentration

Organogelator of 1 mg was added to 1 mL of the solvent. Following the completion of the solution procedure and heating of the solvent to 20 °C below its boiling point, it was cooled in a water bath set at 25 °C and removed and examined to determine whether gelation had occurred after 15–20 min. The concentration at which gelation began was determined as MGC (mg/mL). It was predicted throughout this experiment that the liquid known as the organogelator could not be dissolved in the event that 1 mg of it was not dissolved in 1 mL of liquid (Hanabusa et al., 1996).

### 2.4 Determination of the melting point of gel (*T*_g_)

The organogelator was heated by 1 °C per minute by placing a 0.250 g steel ball into the an bath over gel prepared in a 1 mL organic solvent in an experiment tube with a 10-mm inner diameter. The temperature at which the ball began to fall was determined as the melting point (*T*_g_) of the organogelator (Trivedi et al., 2004). This process was repeated with gels prepared with different concentrations. This value is of great significance in terms of determining whether a organogelator [<1% (w/w)] could enter the supergelator category (Suzuki et al., 2002).

### 2.5 Determination of Δ*H*_g_ gelation enthalpy

Sol–gel transition enthalpy value can be calculated using a Van’t Hoff equation (Eq. 1). The gelation enthalpy in organogelator solvents is determined from the curve of the lines, and then, ln*C*_g_% (organogelator concentration as w%) is transferred to the opposite graph of 1/*T*_g_ (Seo and Chang, 2005). Here, *C*_g_ is organogelator concentration as mol L^-1^, *T*_g_ is the phase transition temperature, and R is the Rydberg gas constant (R = 8.314 J mol^-1^ K^−1^).

$$d\;\ln\left[C_{g}\right]/d\left(1/T_{g}\right)=-\Delta H_{g}/R.$$ (1)

By characterizing the gels that were acquired during the third stage of the investigation, their structure became clearer and SEM methodology was thus applied.

### 2.6 Characterization of gel structure by SEM

Organogelators form organogel with the entanglement of self-forming nanofibers due to their three-dimensional network structure (George and Weiss, 2006). It is possible to observe such nanostructures of organogelators by electron microscopy. For electron microscopic analyses (TEM/SEM), a dry sample gel is generally prepared after it is dried and coated in vacuum. However, a coating method is not used for organogels such as fatty acids created with organic liquids with high boiling point since it cannot give a dry sample (xerogel). The sample preparing systems now in use are provided with a re-precipitation method (Suzuki et al., 2009). Since the organogelators could not dissolve in hexane at room temperature, it is very practical to prepare samples in hexane via rapid precipitation from gels formed by fatty acid esters. The nanoscale network is protected through rapid precipitation, although organogel is damaged in hexane because of shaking.

In the fourth stage of the study, the drug-loading capacity of gels was investigated. The fact that a gel can be loaded with drug is important in terms of their use in releasing activities. Ibuprofen and naproxen were the drugs used.

### 2.7 Drug entrapment capacity of gels

In the present study, ibuprofen (Ib) and naproxen (Npx), known for their pain-killing effect and whose active substance is non-steroidal and anti-inflammatory, were used to determine the drug entrapment capacity of the new organogelators. The amount weighed at a 1:1 ratio with Ib and Npx of organogelator was mixed with 1 mL fatty acid esters in a capped glass test tube (inner diameter: 10 mm); this was heated until the solid substance dissolved. The solution was then allowed to cool and was kept at a constant 25 °C. When a homogenous substance appeared that did not exhibit any gravitational flow when the test tube was turned upside-down, it was anticipated that gelation had occurred. The amount of Ib and Npx was increased until gelation occurred; the maximum Ib and Npx concentration at which gelation occurred was determined as the drug entrapment capacity (Uzan et al., 2016). Entrapping the Ib and Npx capacity of gel was submitted as a result of the sol–gel process.

## 3 Results and discussion

The gelation potential of similar substances has been documented. The novelty of our work is to synthesize two new *C*_2_-symmetric tetraamide compounds and examining their gelation properties by using them as organogelators.

As a result of this study, the gelation potential of organogelator 1 was found to be very high. However, organogelator 2 did not gel with the solvents tested.

### 3.1 Determination of minimum gel concentration

**FIGURE 4:**
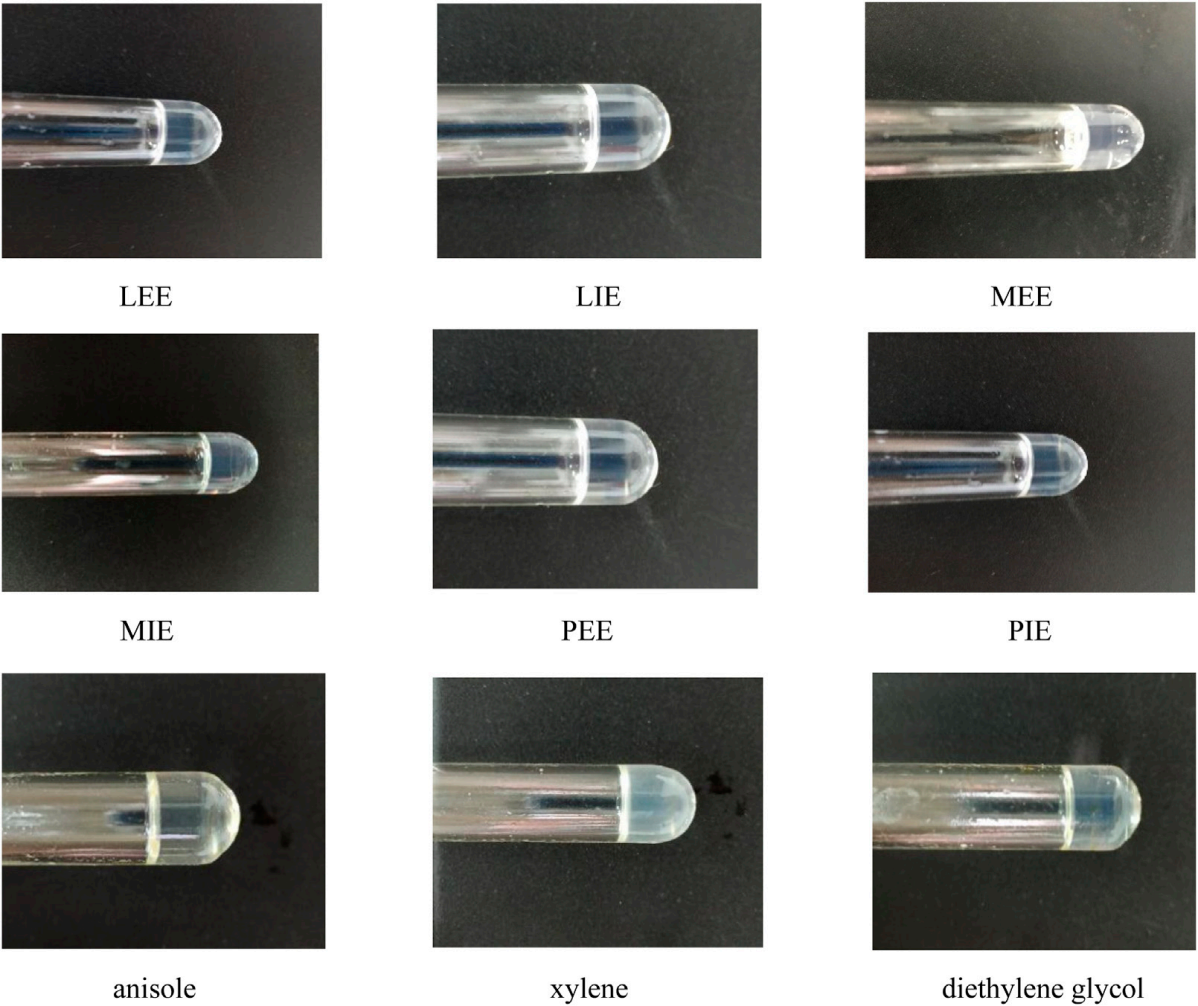
Photographs of organogelator 1 in MGC, LEE, LIE, MEE, MIE, PEE, PIE, anisole, xylene, and diethylene glycol liquids.

As can be seen in Table 1, it was determined that organogelator 1 had gelation ability, transforming most of the liquids into gel. However, it was observed that organogelator 2 could not gel since the organogelator had a solubility problem. In general, gelation in fatty acid esters was found to be superior to common solvents. Photographs of the gels are shown in Figure 4.

**TABLE 1:** MGC of organogelators, mg/mL^-1^.

Solvent	Organogelator 1	Organogelator 2
LEE	4 (tg)	NG
LIE	2 (tg)	NG
MEE	1 (tg)	NG
MIE	5 (tg)	NG
PEE	1 (tg)	NG
PIE	1 (tg)	NG
Xylene	4 (tg)	NG
Anisole	5 (tg)	NG
Diethylene glycol	1 (tg)	NG
N-dodecane	NG	NG
1-Decanol	NG	NG
Liquid paraffin	NG	NG
Toluene	NG	NG
Chloroform	NG	NG

LEE, lauric acid ethyl ester; LIE, lauric acid isopropyl ester.

MEE, myristic acid ethyl ester; MIE, myristic acid isopropyl ester.

PEE, palmitic acid ethyl ester; PIE, palmitic acid isopropyl ester.

tg, transparent gel; NG, no gelation.

### 3.2 Determination of melting point of gel (*T*_g_)

**FIGURE 5:**
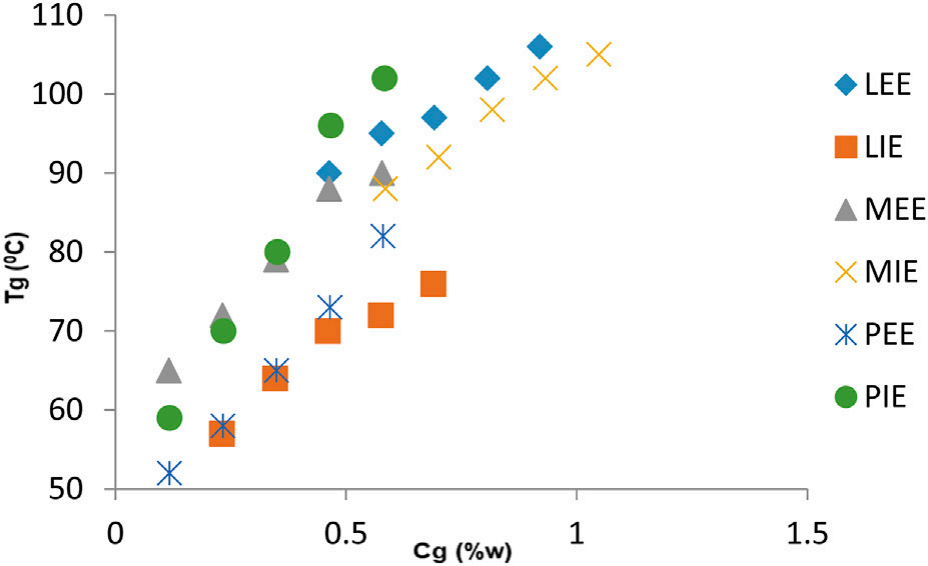
Change in the melting point (*T*_g_) of gels prepared in fatty acid esters of organogelator 1 in relation to gelator concentration *C*_g_ (w%)/*T*_g_.

**FIGURE 6:**
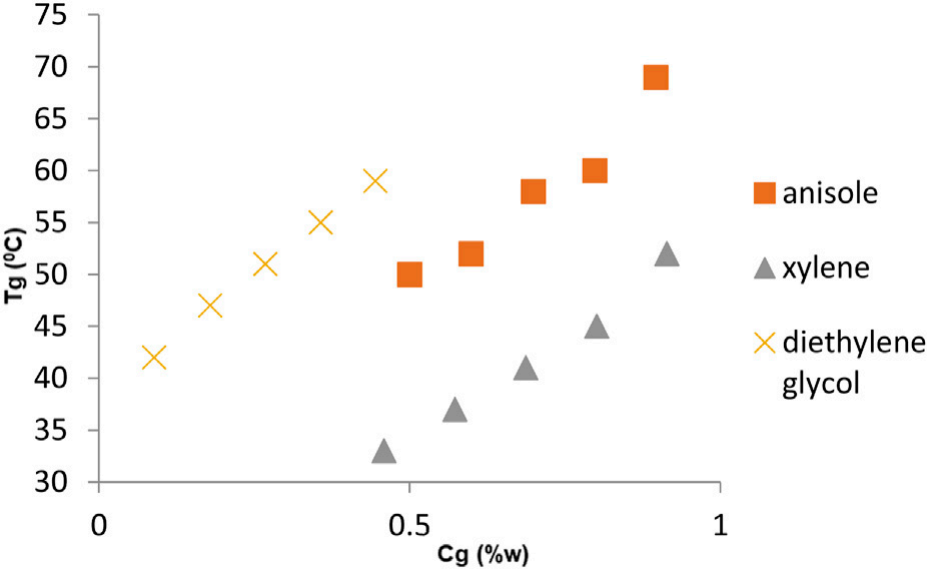
Change in the melting point (*T*_g_) of gels prepared in organic solvents of organogelator 1 in relation to gelator concentration *C*_g_ (w%)/*T*_g_.

Tables 2, 3 show the change in the melting points (*T*_g_) of gels prepared for fatty acid esters (LEE, LIE, MEE, MIE, PEE, and PIE) and common solvents (liquid paraffin, anisole, xylene, toluene, diethyl glycol, and n-dodecane), respectively, against the percentage weight of organogelator in gel matrixes of *C*_g_ (w_g_/w_m_% = % weight in gel matrixes of organogelator); Figures 5, 6 graph these. As is evident from the charts, it was determined that *T*_g_ values of gels prepared with fatty acid esters were higher than *T*_g_ values of gels prepared for common solvents. In addition, it was established that the organogel with the highest *T*_g_ values was the LEE solvent.

**TABLE 2:** Change in melting points (*T*_g_) of gels prepared in fatty acid esters (LEE, LIE, MEE, MIE, PEE, and PIE) of organogelator 1 against % weight of organogelator in gel matrixes of *C_g_ (w_g_/w_m_% = % weight in gel matrixes of the organogelator).

LEE		LIE		MEE		MIE		PEE		PIE	
*C_g_ % w/w	*T*_g_ (^0^C)	*C_g_ % w/w	*T*_g_ (^0^C)	*C_g_ % w/w	*T*_g_ (^0^C)	*C_g_ % w/w	*T*_g_ (^0^C)	*C_g_ % w/w	*T*_g_ (^0^C)	*C_g_ % w/w	*T*_g_ (^0^C)
0.463	90	0.231	57	0.116	65	0.585	88	0.117	52	0.117	59
0.577	95	0.346	64	0.232	72	0.701	92	0.233	58	0.234	70
0.691	97	0.460	70	0.348	79	0.817	98	0.349	65	0.351	80
0.806	102	0.575	72	0.463	88	0.932	102	0.465	73	0.467	96
0.920	106	0.689	76	0.578	90	1.048	105	0.580	82	0.583	102

*Gelator concentration *C*_g_ (as a mol L^-1^) and also given as % weight of organogelators in the gel.

**TABLE 3:** Change in melting points (*T*_g_) of gels prepared in common solvents (anisole, xylene, and diethyl glycol) of organogelator 1 against % weight of the organogelator in gel matrixes of *C_g_ (w_g_/w_m_% = % weight in gel matrixes of the organogelator).

Anisole		Xylene		Diethylene glycol	
*C_g_ % w/w	*T*_g_ (^0^C)	*C_g_ % w/w	*T*_g_ (^0^C)	*C_g_ % w/w	*T*_g_ (^0^C)
0.500	50	0.459	33	0.089	42
0.599	52	0.573	37	0.179	47
0.699	58	0.687	41	0.268	51
0.798	60	0.801	45	0.357	55
0.896	69	0.914	52	0.445	59

* Gelator concentration *C*_g_ (as a mol L-1) and also given as % weight of organogelators in the gel.

The change between w% and *T*_g_ for gels in fatty acid esters (Figure 5) and common solvents (Figure 6) of organogelator 1 is presented collectively. It was thus observed that the *T*_g_ values of organogelators increased in relation to the concentration of gel. Furthermore, in comparison to fatty acid esters, it was determined that *T*_g_ values of gels prepared in LEE, LIE, and MEE were close to each other, while gels prepared with PIE had lower *T*_g_ values. When the *T*_g_ values of gels prepared in common solvents were examined, the lowest values were for those prepared with xylene.

### 3.3 Determination of Δ*H*_g_ gelation enthalpy

**FIGURE 7:**
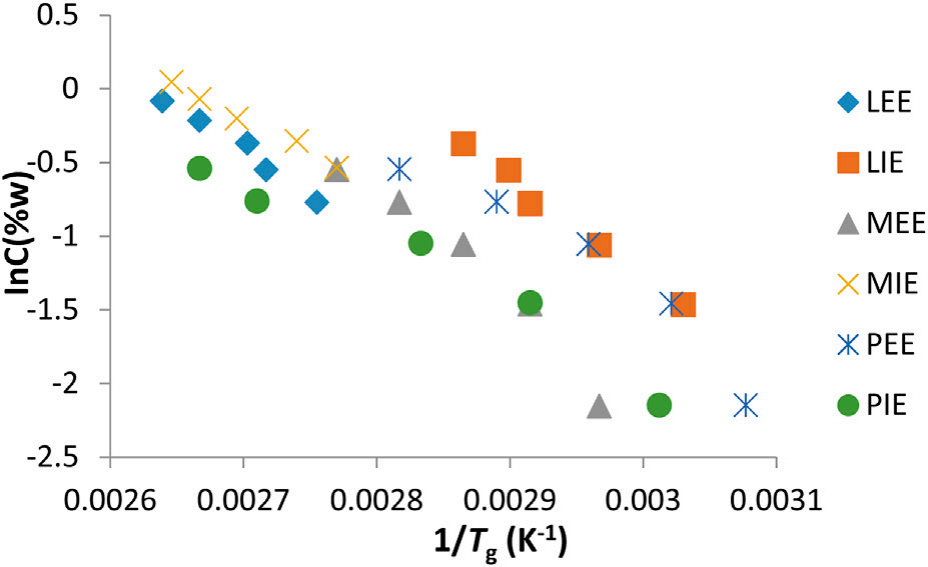
an’t Hoff graphs of gels prepared in fatty acid esters of organogelator 1.

**FIGURE 8:**
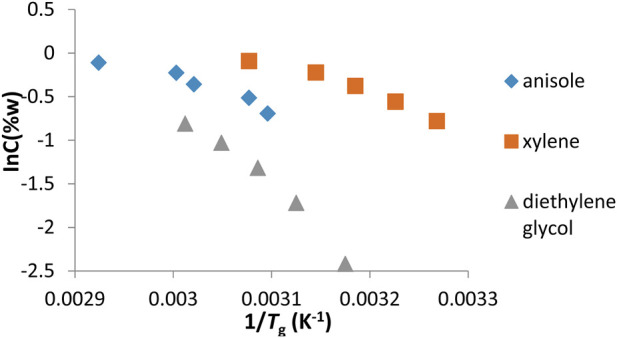
an’t Hoff graphs of gels prepared in organic solvents of organogelator 1.

The change of gels prepared for fatty acid esters (LEE, LIE, MEE, MIE, PEE, and PIE) and common solvents (anisole, xylene, and diethyl glycol) of organogelator 1 against ln*C*_g_ (w%) was transferred to the graph shown in Figures 7, 8 respectively. From the curve of the lines obtained, the gelation enthalpy Δ*H*_g_ is determined and is given in Table 4. A high Δ*H*_g_ (gel–sol transition enthalpy) value indicates that it provides a stable network structure. As can be seen in Table 4, diethylene glycol gel had the highest Δ*H*_g_ value with 81.717 kj mol^-1^ of all solvents, while the anisole gel had the lowest Δ*H*_g_ value with 26.996 kj mol^-1^. Of all fatty acid esters, the solvent MEE had the highest Δ*H*_g_ value with 60.100 kj mol^-1^, while the solvent PIE had the lowest Δ*H*_g_ value with 36.128 kj mol^-1^.

**TABLE 4:** Gel−sol transition enthalpies (Δ*H*_g_, kJ mol^−1^) of organogelator 1 calculated from Van’t Hoff Eq. 1.

Solvent	∆*H*_g_ (kJ mol^−1^)
LEE	49,690
LIE	55,306
MEE	60,100
MIE	37,276
PEE	48,966
PIE	36,128
Anisole	26,996
Xylene	30,090
Diethylene glycol	81,717

### 3.4 Characterization of gel structure by SEM

**FIGURE 9:**
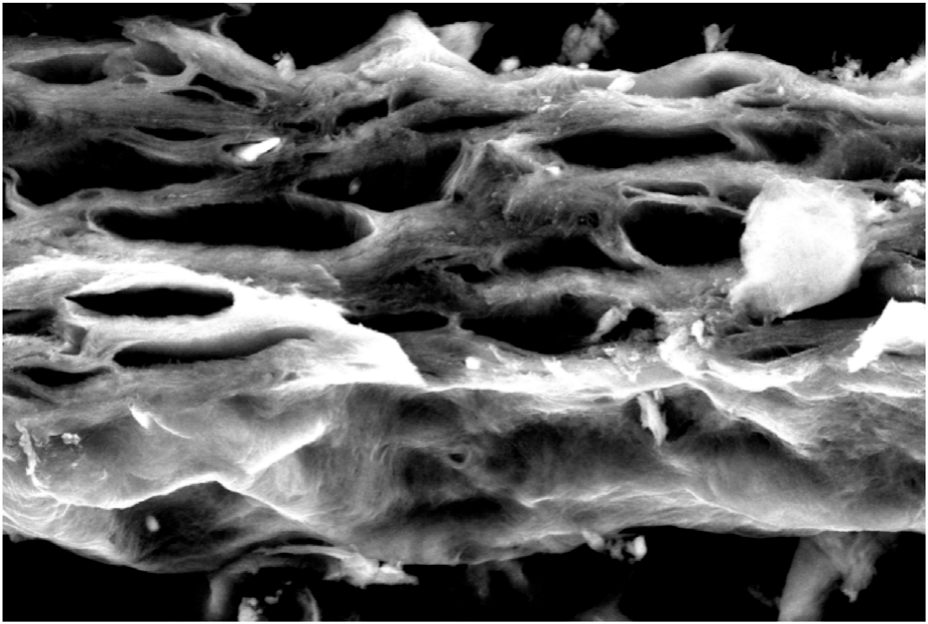
SEM image of the gel prepared in diethylene glycol of organogelator 1.

**FIGURE 10:**
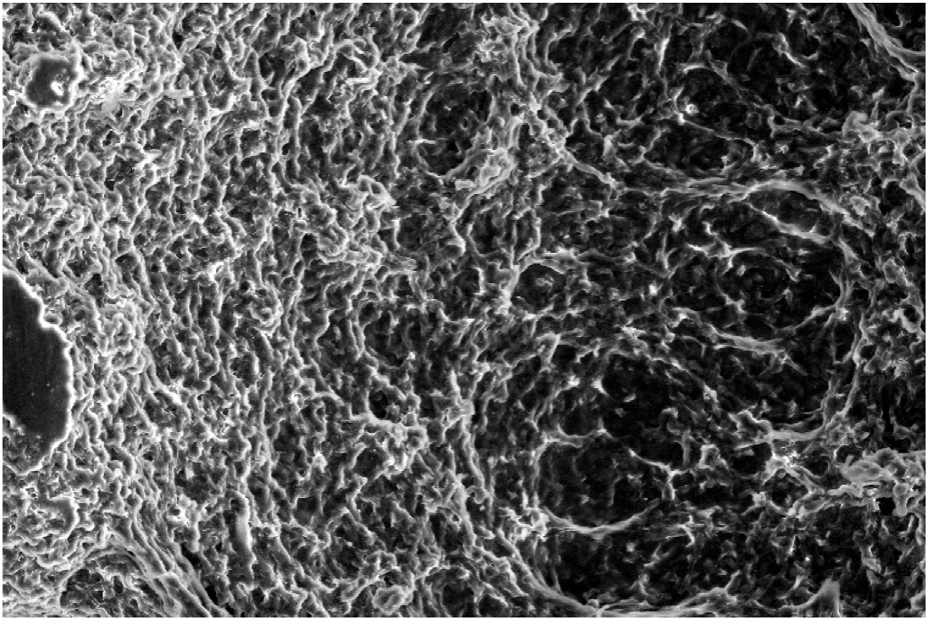
SEM image of the gel prepared in PIE of organogelator 1.

**FIGURE 11:**
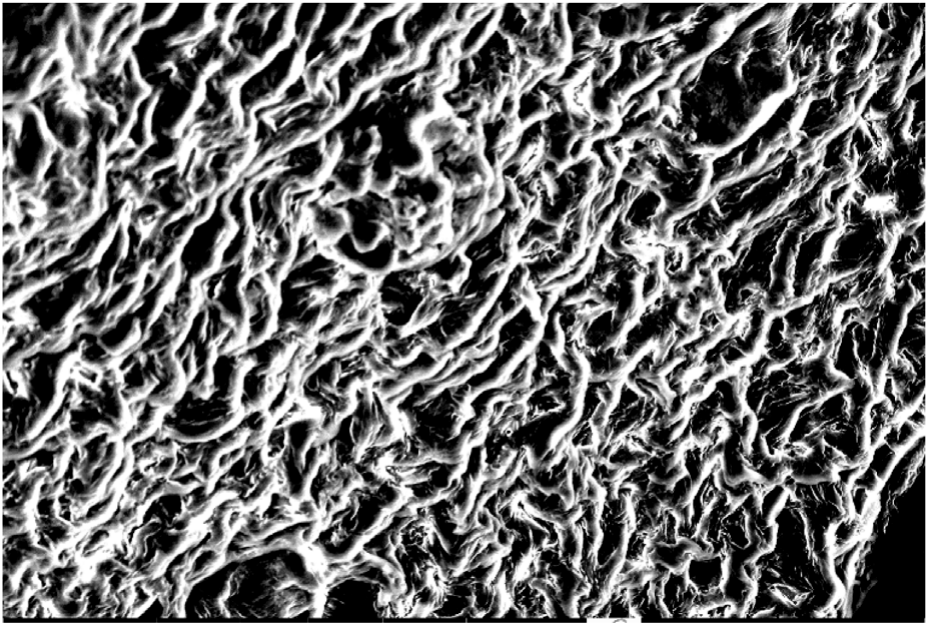
SEM image of the gel prepared in LIE of organogelator 1.

The solvent was removed from the gel structure prepared in 1 mL solvent of organogelator by hexane extraction. At this stage, the organogels were mixed strongly, then hexane was added, and the white precipitate was filtered and washed with hexane. It was next kept under vacuum with freeze-drying for 24 h, and a SEM image of the organogelator obtained as xerogel was taken (Figures 9–11). The SEM images show the helix bundle self-formed from nanofibers.

### 3.5 Drug entrapment capacity of gels

**FIGURE 12:**
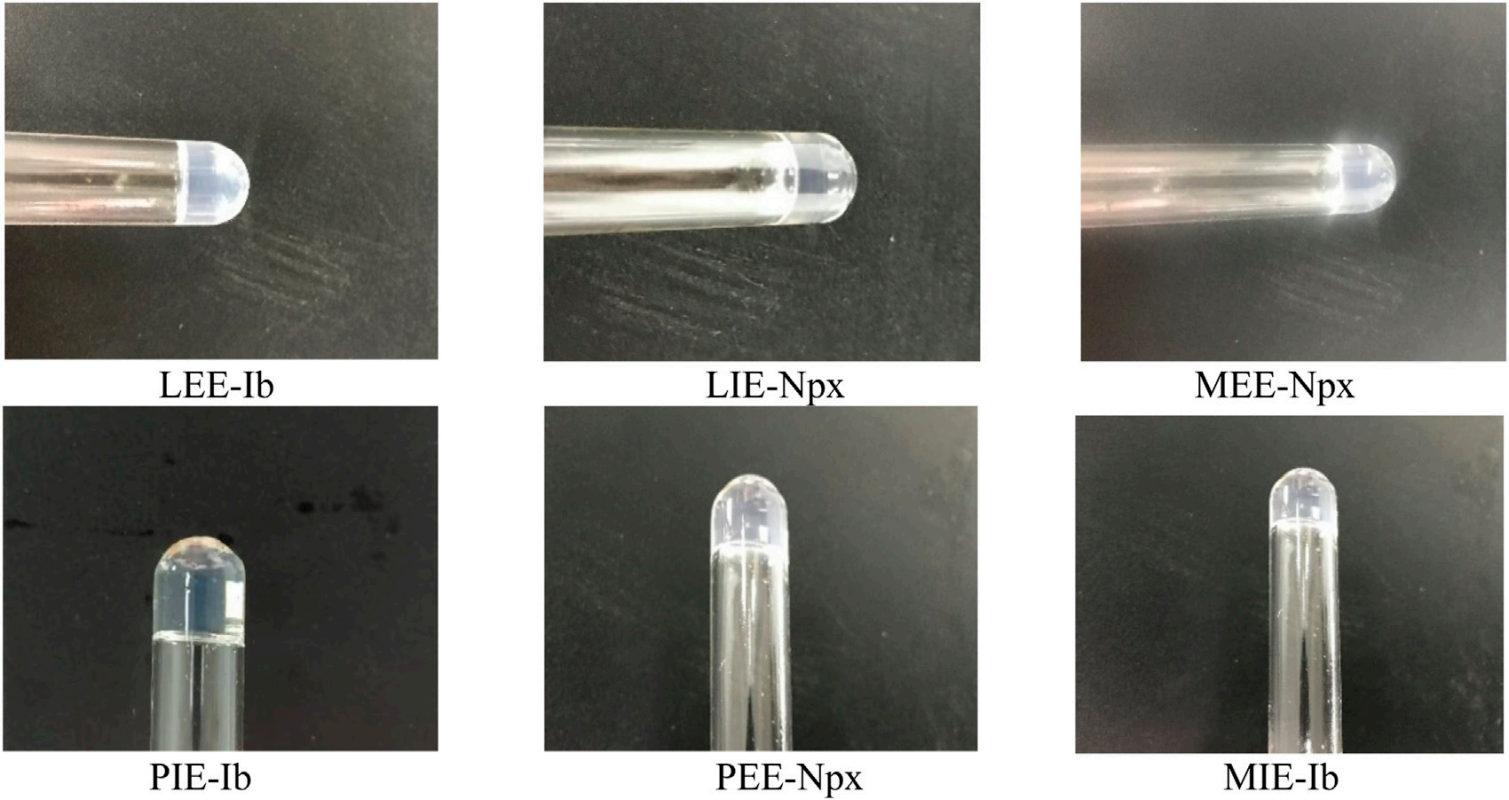
Photographs of gels LEE, LIE, MEE, MIE, PEE, and PIE solvents of organogelator 1 given with Ib and Npx.

Ibuprofen and naproxen were gradually loaded with 6.1 mg of organogelator 1 onto gels prepared with 1 mL fatty acid esters (LEE, LIE, MEE, MIE, PEE, and PIE) until its gel structure broke down (Figure 12). The maximum drug entrapment capacities of the gels were thus determined (w/w%-Drug/Organogelator*100). The experimental data obtained are given in Table 5. The Ib- and Npx-loading capacity of the gels formed from the fatty acid esters of organogelator is high. For this reason, it can be used in drug release work. It can be observed that the structure of gelator–gelation dual liquids and the kind of drug affect the drug-loading capacity. In general, it was determined that more Npx can be loaded than Ib. This is important in realizing the drug in a controlled way. It was determined that the drug-loading capacity of gels prepared with PEE and PIE is higher than those prepared with LIE. This demonstrates that the drug-loading capability of a fatty acid ester chain rises with its length. Furthermore, it was noted that, in isopropyl esters of fatty acid esters, Ib- and Npx-loading capabilities are nearly identical. The highest drug-loading capacity is in the gel prepared with Ib of PIE.

**TABLE 5:** Ibuprofen- and naproxen-loading capacity of gels with fatty acid esters of organogelator 1.

Solvent	Ibuprofen	Naproxen
LEE	1.59	1.81
LIE	1.83	1.83
MEE	2.04	2.26
MIE	2.06	2.06
PEE	2.71	2.92
PIE	2.94	2.72

## 4 Conclusion

The synthesized organogelators are more advantageous than polymeric gelators in terms of their ease of removal from the body as they have low molecular mass. In addition, the low amount of organogelator required for gelation is an advantage for cosmeceutical ingredients. The prepared organogels are biocompatible since the solvents used in gelation are those used in the drug industry, such as liquid paraffin and fatty acid esters, since the structure of organogelators consists of amino acid structures such as *L*-isoleucine, and since they contain functional groups such as peptide and amide, which the human body recognizes. Organogelator 1 produced gel with similar concentrations to most solvents widely used in the cosmetics and drug industries. Organogelator 2 had solution problems, so it could not produce gel in the experimental solvents. The side group possessed by organogelator 2, which set it apart from organogelator 1, is assumed to be the cause of this. Since the fatty acid esters chosen as gelation liquids were liquids used in the drug industry, ibuprofen and naproxen were loaded with the gels that these liquids created, and their loading capacity was investigated. The organogels created have the potential to be employed as drug-carrying systems, and organogelator 1 was found to have excellent drug-loading capacity.

## Data Availability

The original contributions presented in the study are included in the article/Supplementary Material; further inquiries can be directed to the corresponding author.
