# Supplementary material for: Investigation of the synthesis, gelation potential, and drug-loading capacities of two novel amides
Source: Front Chem. 2024 May 10;12:1369542. doi: 10.3389/fchem.2024.1369542 (PMC11117075; doi:10.3389/fchem.2024.1369542)
Supplement: Supplementary file 1 [file DataSheet1.PDF]

Investigation of synthesis, gelation potentials and drug loading capacities of two novel amides

Deniz BARIŞ CEBE[1,*], Elif KÖTEKOĞLU[1]

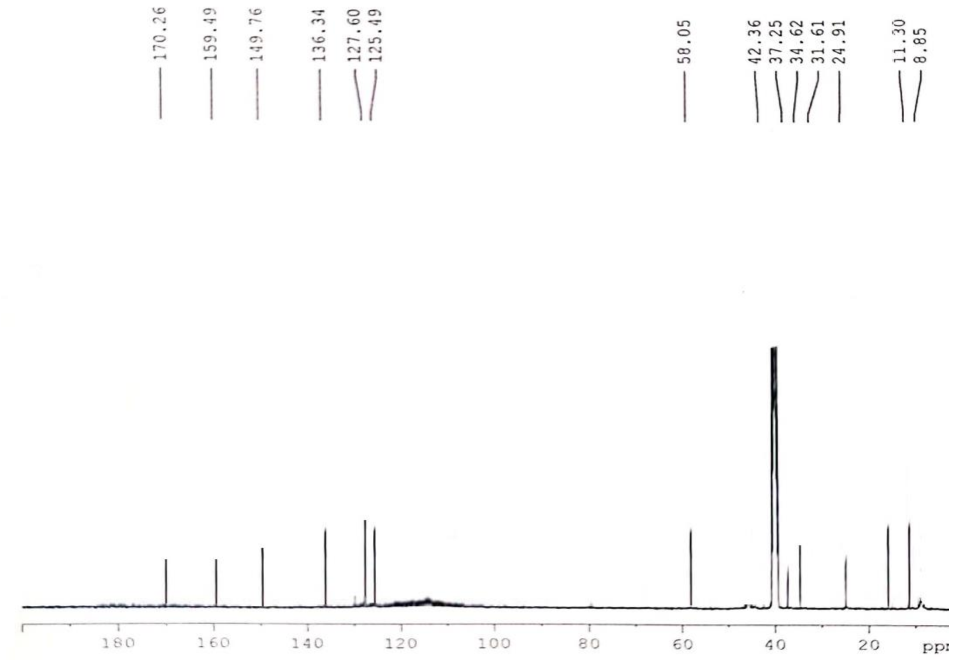

The 100 MHz ^{13}C NMR spectra of the organogelator 1

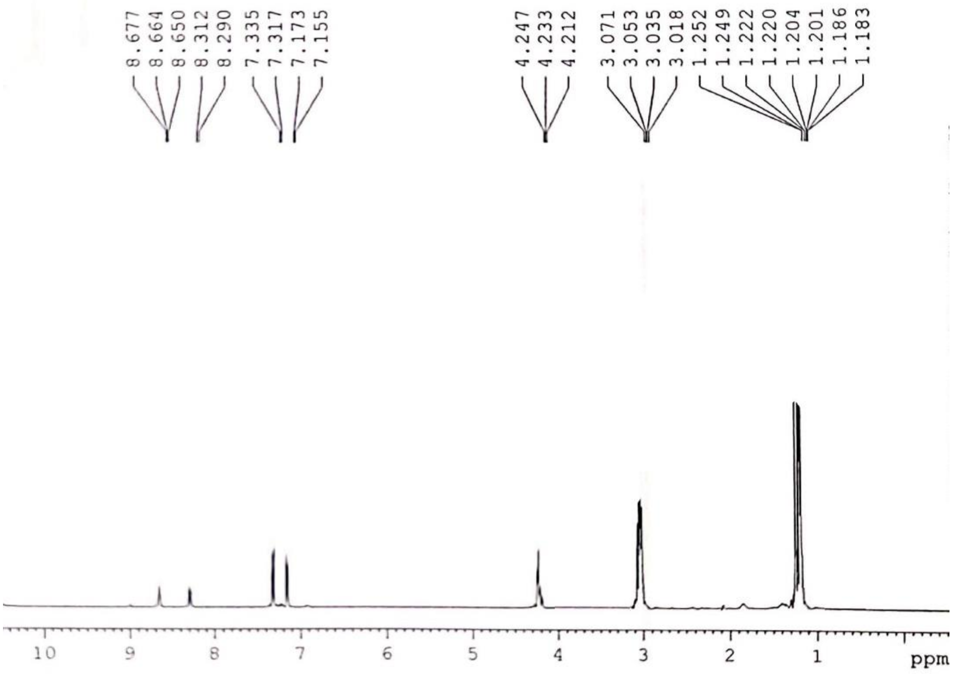

The 400 MHz ^{1}H NMR spectra of the organogelator 1

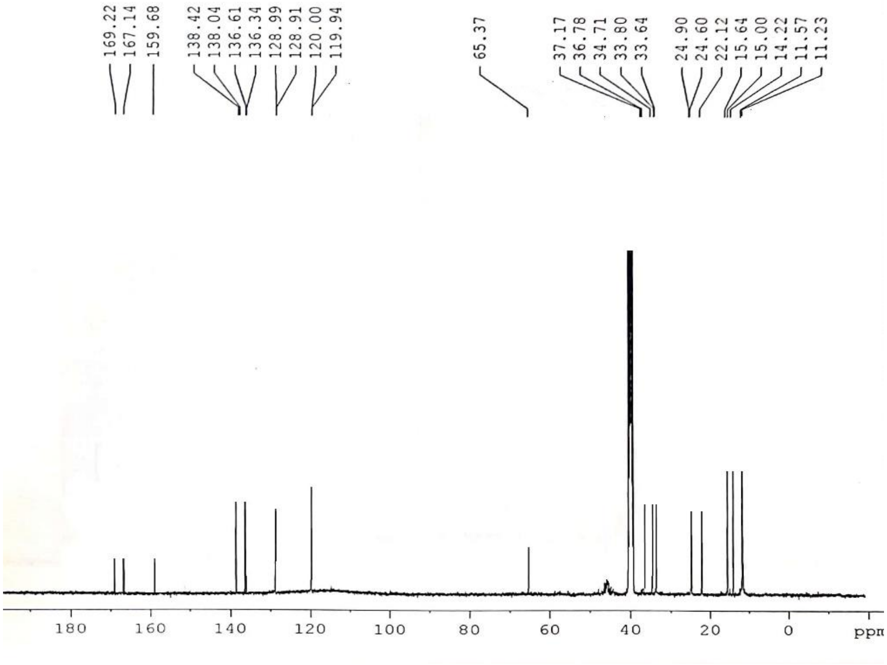

The 100 MHz ^{13}C NMR spectra of the organogelator 2

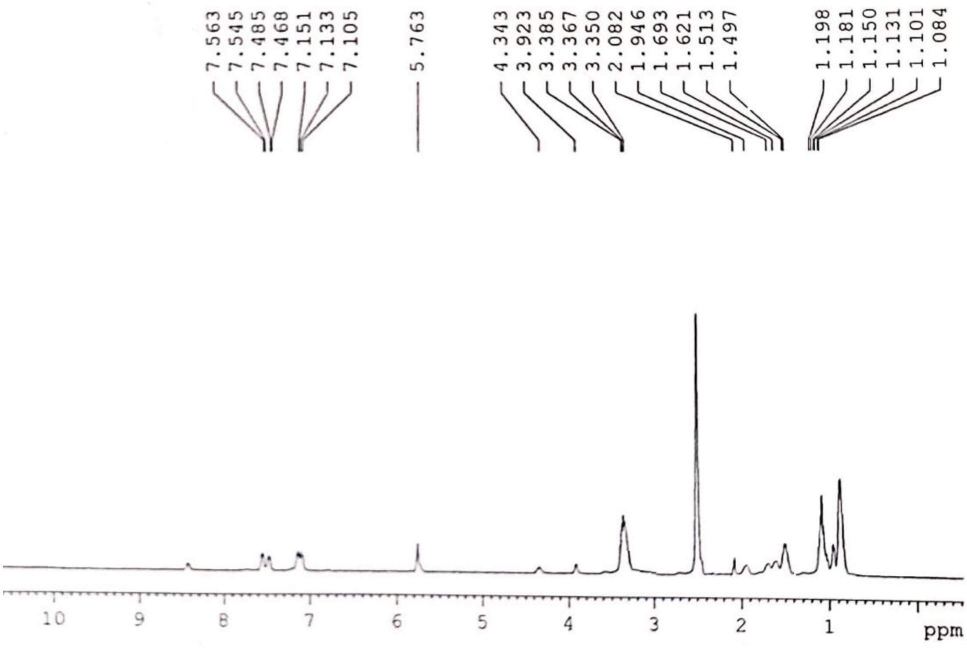

The 100 MHz ^{1}H NMR spectra of the organogelator 2

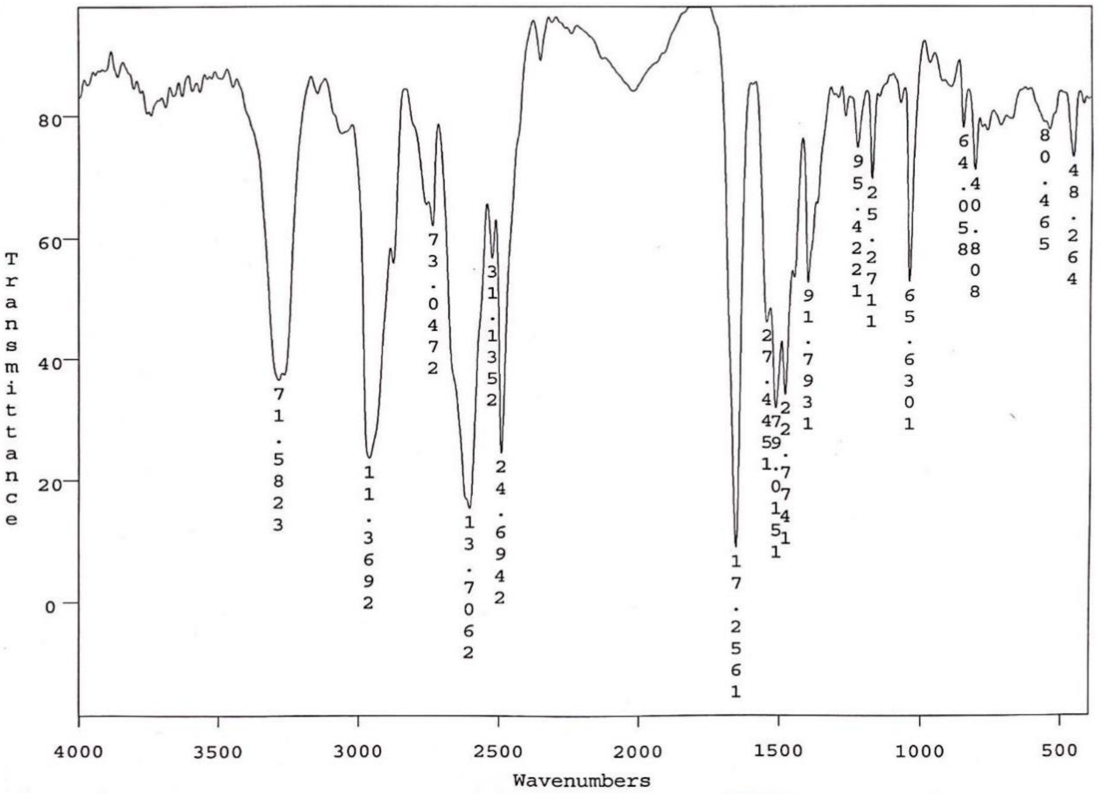

The FTIR spectra of the organogelator 1

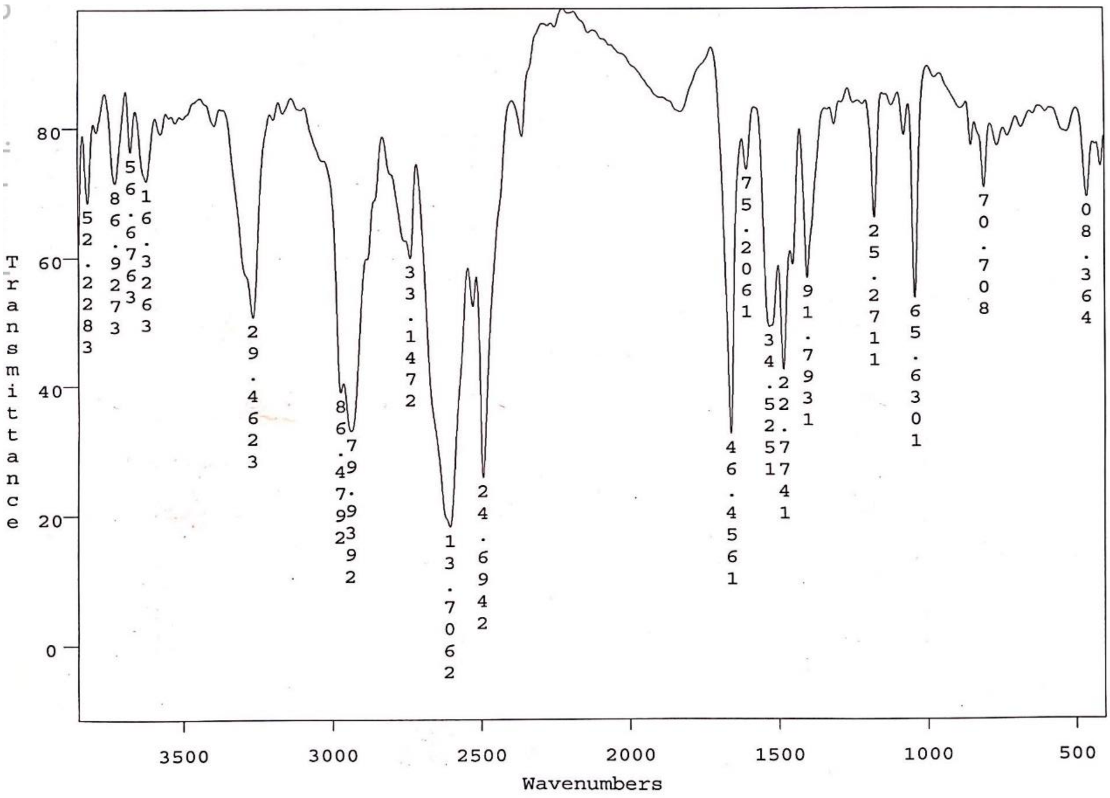

The FTIR spectra of the organogelator 2
